# 

**Published:** 1875-08

**Authors:** 


### CONTENTS:

### ORIGINAL COMMUNICATIONS.

ARTICLE I.—The Causes of Irregularity in the Development of the Teeth. By N. W. Kingsley, D. D. S., M. D. .......... 145
ARTICLE II.—Memoir of Dr. George Sydney Jones. By W. H. Morgan, D. D. S., M. D. .......... 163
ARTICLE III.—Georgia State Dental Society .......... 164
American Academy of Dental Science .......... 167

### SELECTED ARTICLES.

ARTICLE IV.—Treatment of Deciduous Teeth. By Henry S. Chase, D. D. S., M. D. 167

ARTICLE V.—Food for Infants During Dentition. By Dr. B. F. Dawson .......... 174

ARTICLE VI.—A New Motor .......... 176

ARTICLE VII.—The Dental Profession in France. By Mordaunt Stevens, M. R. C. S., D. D. S. .......... 179

ARTICLE VIII.—An Easy Method of Removing a Part of the Inferior Dental Nerve within the Lower Jaw. By John T. Hogden, M. D. .......... 181

### EDITORIAL, ETC.

Summer Sessions .......... 182

A Sad Occurrence—The Murder of Two Dentists .......... 183

### MONTHLY SUMMARY.

Billroth on the Repair of Tissues .......... 186

Camphorated Chloral in Neuralgia .......... 187

Consanguineous Marriages .......... 188

The Teeth as Sexual Organs .......... 188

Medical and Dental Graduates .......... 188

Action of Mercury upon the Blood .......... 189

Epistaxis Arrested by Compressing Facial Arteries .......... 189

New Method of Arresting Hemorrhage .......... 190

Solubility of Salicylic Acid .......... 190

Structure of Tumors as Related to their Character .......... 191

Narcotizing Horses .......... 191

Women-Doctors in Russia .......... 191

Relation of Alcohol to Animal Heat and Muscular Power .......... 192

Snow as a Hæmastatic .......... 192

Warts .......... 192

Nitrous Oxide in Tuberculosis .......... 192

A Convenient Local Anodyne .......... 192

